# False-Positive and False-Negative MRD Results in Children with Acute Lymphoblastic Leukemia: Navigating Between Scylla and Charybdis (Brief Review and Clinical Experience)

**DOI:** 10.3390/children12070860

**Published:** 2025-06-30

**Authors:** Yulia S. Korkina, Timur T. Valiev, Natalia A. Batmanova, Mikhail V. Kiselevskiy, Irina Z. Shubina, Kirill I. Kirgizov, Svetlana R. Varfolomeeva

**Affiliations:** 1 Research Institute of Pediatric Oncology and Hematology, FSBI “N.N. Blokhin National Medical Research Center of Oncology” of the Ministry of Health of Russia, Kashirskoye Sh.23, Moscow 115522, Russia; juliaskomorokhova@mail.ru (Y.S.K.); timurvaliev@mail.ru (T.T.V.); batmanova_nataly@mail.ru (N.A.B.);; 2 Research Institute of Experimental Diagnostics and Therapy of Tumors, FSBI “N.N. Blokhin National Medical Research Center of Oncology” of the Ministry of Health of Russia, Kashirskoye Sh.24, Moscow 115522, Russia

**Keywords:** minimal residual disease, false-positive MRD, false-negative MRD, flow cytometry, acute lymphoblastic leukemia, children, genomic methods

## Abstract

Background/Objectives: Acute lymphoblastic leukemia (ALL) is the most common malignant disease in children. Contemporary antitumor treatment protocols provide long-term survival rates in over 90% of patients with ALL. High effectiveness of the treatment has been achieved as a result of chemotherapy optimization, use of targeted drugs, up-to-date genetic information, and detection of minimal residual disease (MRD). Current highly sensitive methods for MRD detection have advantages and disadvantages, and the challenge is to distinguish between false-positive and false-negative tests. Methods: A comprehensive search through MEDLINE, PubMed, Scopus, and ScienceDirect using the MRD-related keywords was performed, and included a final set of 72 academic articles. Results: At present, flow cytometry for MRD detection provides the necessary sensitivity of 10^−4^ and allows for reliable prediction of ALL dynamics and effective therapeutic strategies. However, even multicolor flow cytometry (MFC) cannot avoid cases of false-positive or false-negative results. Highly sensitive and productive genomic methods in addition to MFC may enhance the accuracy of MRD evaluation. On the other hand, overwhelming efforts to reach the highest sensitivity of the detection methods may lead to the detection of clinically insignificant manifestations of minimal residual disease and, subsequently, to unjustified escalation of antitumor therapy. Conclusions: The necessary ground for an adequate sensitivity of the MRD detection methods could ensure the fine line between false-positive and false-negative MRD results in patients with childhood ALL to develop an appropriate therapeutic strategy.

## 1. Introduction

Despite the fact that acute lymphoblastic leukemia (ALL) is the most common malignant disease in children, current antitumor treatment protocols provide long-term survival rates of over 90% for patients. However, disease relapses are still the main problem leading to poor outcomes and occur in approximately 20% of children with ALL [1]. High effectiveness of the treatment has been achieved as a result of chemotherapy optimization, use of targeted drugs, up-to-date genetic information, and detection of minimal residual disease (MRD) [2].

Traditionally the researchers use multicolor flow cytometry (MFC), real-time quantitative polymerase chain reaction (RT-qPCR), or digital PCR for MRD detection; recently they have introduced a novel method of next-generation sequencing (NGS) for MRD detection [1,2,3]. MRD results are the basis for risk stratification as well as for treatment strategy [4]. A number of studies showed that a negative MRD test was associated with a low risk of ALL relapse, while a positive MRD test correlated well with a high risk of relapses [5,6,7,8,9,10]. Clinical practice protocols widely include MRD detection in patients with hemoblastoses [4,11]. MRD assessment is important not only for stratification of the risk of disease recurrence but also for the choice of treatment strategy, in particular, when choosing chemotherapy, hematopoietic stem cell transplantation, or CAR-T therapy [12,13]. However, the interpretation of MRD results as a prognostic or predictive factor is associated with a number of limitations related to the reproducibility and accuracy of the method used. The lack of clear standards and different methods of MRD assessment can lead to false-positive and false-negative results [14,15]. In addition, it should be noted that despite modern, highly sensitive methods for MRD detection, some patients with negative MRD develop a relapse (false-negative results), while some patients with positive MRD have no disease progression (false-positive results); therefore, the search for adequate methods for MRD assessment that do not lead to underdiagnosis or overdiagnosis is still an urgent problem.

Therefore, this review focuses on the advantages and disadvantages of up-to-date methods for MRD detection and the importance of distinguishing between false-positive and false-negative tests.

## 2. Methods

A comprehensive search was performed through MEDLINE, PubMed, Scopus, and ScienceDirect using the following keywords: “MRD”, “MRD and MFC”, “MRD and genomic methods”, “MRD and qPCR”, “MRD and next-generation sequencing”, “MRD and false-positive”, “MRD and false-negative”, and “MRD and children with ALL” in the published papers over the last decade. Reviews, systematic reviews, meta-analyses, clinical trials, and randomized clinical trials were selected, particularly those that focused on MRD diagnostic approaches in patients with childhood ALL. The final set included 72 articles related to the topics of interest.

## 3. Results

### 3.1. Minimal Residual Disease

In 1993, Potter et al. defined minimal residual disease as a “disease occurring at a subclinical level and beyond detection by conventional methods of assessment” [16]. Identification of the most sensitive prognostic factors for predicting a relapse was very important for “risk-adjusted therapy” [2]. MRD estimation during the treatment of various hematological malignancies has important prognostic implications regarding disease relapse and personalized therapy for children with ALL [17].

At the same time, a binary approach to MRD detection determines only a negative or a positive result, which does not allow accurate assessment of the risk of recurrence and, accordingly, justify the choice of treatment strategy [18,19]. This approach makes it difficult to compare the risks of recurrence in patients with high and low MRD levels [20]. In accordance with the ALL-IC BFM 2002 protocol, MRD was assessed by PCR in peripheral blood on day 8 and the number of blasts in the bone marrow on day 15. Following these results, the patients were divided into risk groups. According to the ALL-IC BFM 2009 protocol, patients were stratified on the basis of MRD detection by MFC on day 15 of the induction phase [21]. The expediency of stratifying patients by risk groups was confirmed in a number of studies. In particular, Samardzić-Predojević et al. showed that patients with low MRD (0.1–10%) had a 5-year overall survival (OS) and event-free survival (EFS) of 89.5% and 91%, respectively, while OS and RFS in patients with MRD > 10% were just 80% [22]. In a Chinese multicenter retrospective clinical trial, 1164 patients with ALL were divided into three groups after MRD detection on day 15 of induction therapy: MRD < 0.1%, MRD 0.1–10.0%, and MRD ≥ 10.0%, and on day 33 they were divided into MRD < 0.01%, MRD 0.01–1.0%, and MRD ≥ 1.0% groups. The 5-year OS in the groups MRD < 0.1%, MRD 0.1–10.0%, and MRD ≥ 10.0% were 95.4 ± 1.0%, 93.3 ± 1.1%, and 85.4 ± 2.9%, respectively, and RFS—93.2 ± 1.6%, 90.8 ± 1.4%, and 78.9 ± 4.3%, respectively. On day 33, the 5-year RFS in the MRD 0.01–1.0% group was the lowest among the three groups (91.4 ± 1.2%, 84.5 ± 3.2%, and 87.9 ± 5.1%, respectively). The authors made a conclusion that the MRD determination on day 15 of the induction therapy guarantees a more accurate stratification of patients into prognostic risk groups [23]. “Risk-adjusted therapy” based on MFC-MRD allows increasing the survival rate of patients with ALL [24]. Othus et al. proposed extending the options for MRD assessment by subdividing it into four variants: (1) true-positive, (2) true-negative, (3) false-positive, or (4) false-negative, but the authors did not provide the results of such a stratification [25]. The accuracy of MRD detection is affected by the frequency of testing and the timing of the follow-up, the location of sampling, and the number of bone marrow samples. Repeated MRD detection can reduce false-positive and false-negative results. However, repeated MRD assessments may present contradictory data [26]. A consensus document of the European LeukemiaNet MRD Working Party (ELN) recommends an MRD level of 0.1%, which is informative for 500,000–1,000,000 cell counts [27,28]. However, MRD < 0.1% can also have a prognostic value [29]. Clinical importance is considered to be significant with an MRD level of 0.01%, i.e., one transformed cell per 10,000 mononuclear cells [30]. Clinical studies demonstrated that high levels of MRD in bone marrow (10^−2^–10^−3^) before hematopoietic stem cell transplantation (HSCT) were associated with a high probability of leukemia recurrence after HSCT (80%), while low (10^−5^) and undetectable MRD levels were associated with a recurrence probability of only 25–30% after HSCT [31]. Various methods are used for MRD detection that focus on the immune phenotype or cytogenetic and/or molecular abnormalities, which are different in sensitivity and specificity of assessment; however, each method has both advantages and disadvantages [25] (Table 1). Therefore, insufficient specificity of MRD detection methods may lead to false-negative results. On the other hand, current trends for excessive enhancement of the sensitivity of the methods may also become the cause of false-positive results. At present, there is no “gold standard” that allows researchers to evaluate MRD with 100% probability. The abovementioned considerations indicate the importance of a detailed analysis of modern detection methods for a comparative assessment of their advantages and disadvantages, which may affect the accuracy of MRD detection and, consequently, patient treatment strategies.

### 3.2. Methods for MRD Detection

Multicolor flow cytometry, polymerase chain reaction, and next-generation sequencing (NGS) are generally used to evaluate MRD levels. Although these modern methods of MRD assessment have their limitations, they are approximately 100 times more sensitive than standard pathomorphological tests (Table 1). In relation to MRD evaluation, immunological methods (MFC-MRD) and molecular methods (qPCR-MRD), as well as NGS or dPCR, are used to count blast cells beyond the light-optical level [16,17,18,19]. MRD detection on certain days of treatment is a common current criterion for patient stratification into risk groups [1]. These methods and their combinations for MRD detection used in clinical practice can hardly achieve accurate prediction of the recurrence risk.

The MFC-MRD is an easily accessible, straightforward, cost-effective, and widely used method for MRD detection that provides rapid results. The use of four-color staining allows MRD measurement at the appropriate diagnostic level of 0.01%. This method enables direct quantitative MRD determination and the assessment of potential immunotherapy markers. There are two main approaches to evaluate MRD by flow cytometry. The first one is the determination of leukemia-associated immunophenotypes (LAIPs). It is based on the immunophenotype of blasts at the time of disease diagnosis. This approach identifies aberrations to reveal leukemic blasts. Another one, the «difference from normal (DfN)» approach, detects immunophenotypic shifts and aberrations compared to expected maturation patterns in the bone marrow. It can be used in the absence of a diagnostic sample and is especially useful in cases of clonal shift; however, it requires an expert level of knowledge of normal maturation patterns. Nevertheless, standard MFC-MRD has limited sensitivity and is prone to false-negative results, which often occur in samples where the immunophenotype of leukemic blasts is significantly similar to that of hematogones or normal B-cells [32].

The main disadvantage of classical MFC-MRD is the lack of standardization of immunophenotyping protocols and panels of selected antibodies, which significantly vary between centers. To improve the accuracy of MFC-MRD, it is recommended to include markers that can maximally differentiate residual blasts from other hematopoietic cell elements. Leukemic cells of many patients with B-ALL demonstrate aberrant expression of B-cell markers such as CD19, CD22, CD10, CD20, CD34, and CD4. In addition to the aberrant expression of B-cell maturation markers, non-B-cell markers may also be expressed in B-ALL. CD13 and CD33, myeloid lineage markers (not expressed on normal B-cells), are expressed in 40% of patients with B-ALL [33]. The concept of aberrant expression of B-cell and non-B-cell markers has been defined as the expression of LAIPs, and LAIPs were observed in 92% of patients with B-ALL. Recent advances have demonstrated the significance of the new markers such as CD58, CD81, CD304, CD73, CD66c, and CD123 that enhance MRD identification [22]. The UKALL Flow MRD group has developed a four-color antibody panel using CD34/CD19/CD10 as a foundation, supplemented with eight different maturation and aberrancy markers (CD13, CD20, CD22, CD33, CD38, CD45, CD58, and KORSA) [34].

Although these MRD panels were effective, it was still complicated to reveal the difference between normal B-cells and B-ALL cells, especially in the samples with a high level of normal/regenerating B-cells (e.g., at the end of induction therapy or after stem cell transplantation) [35]. Therefore, MFC-MRD needs further improvements to better differentiate between normal and malignant B-cells. Coustan-Smith et al. introduced 22 new markers (CD44, BCL2, HSPB1, CD73, CD24, CD123, CD72, CD86, CD200, CD79b, CD164, CD304, CD97, CD102, CD99, CD300a, CD130, PBX1, CTNNA1, ITGB7, CD69, CD49f), which were expressed in 81.4% of ALL cases. The expression of some markers was associated with genetic anomalies, potentially useful for assessing B-ALL [36]. To enhance the sensitivity of MFC-MRD, the EuroFlow consortium recommended using eight to nine colors for immunostaining. New markers and analysis of a large number of cells made MFC-MRD equally sensitive to PCR [37]. Another study described an optimized 12-color antibody panel for the flow cytometry test designed for the diagnosis of B-ALL and MRD monitoring in a single tube [38]. This method reliably differentiated hematogones and B-ALL cells using two specific markers: CD43 and CD81. These and other markers (for example, CD73, CD66c, and CD49f) improved the specificity of detecting B-ALL cells. This approach was based on a dual strategy of DfN/LAIPs. The reliability of the MRD monitoring was confirmed by a strong correlation (r = 0.87) with qPCR results [38].

However, MFC-MRD has many advantages as compared with PCR-MRD, such as easy performance, labor effectiveness, obtaining rapid results, economic efficiency, availability, and applicability. To avoid false-negative results, MFC-MRD allows the simultaneous assessment of normal hematopoietic elements and the quality of the bone marrow sample. Recent studies have highlighted the significance of the new markers (e.g., CD58, CD81, CD304, CD73, CD66c, and CD123) that enhance MRD identification. The eight-color EuroFlow consortium protocol can achieve a sensitivity of up to 10^−5^ if a sufficient number of cells are collected. It is comparable to PCR-based methods [34]. Currently, most assays used for MRD detection are based on the identification and tracking of populations with phenotypic or genomic aberrancies that correspond to leukemic blasts [34,35,36,37,38,39,40,41,42,43,44]. Numerous clinical studies have shown that detecting leukemia-specific markers with MFC-MRD allows for the prediction of outcomes in patients with ALL. MFC-MRD can be used more efficiently than molecular methods since flow cytometry is already available in most cancer centers worldwide [22,23].

However, it should be noted that an excessive increase in MFC sensitivity can lead to false-positive results. In particular, with the sensitivity of the MFC-MRD test of 0.01% and the recommended number of 5 × 10^6^ bone marrow cells to be evaluated, the MFC method is accurate enough to detect 50 target blasts to determine a positive MRD [18]. Enhanced sensitivity of the method by an order up to 0.001% will decrease the threshold of positive MRD to five blast cells. Given the lack of the standardization of MFC results, the inevitable subjectivity of operators, and the limit of MFC measurement accuracy, the benefit of increased MFC sensitivity seems doubtful, since it can lead to false-positive MRD results. On the other hand, false-negative MRD following MFC-MRD assessment can occur as a result of different bone marrow sampling and low cell numbers after previous chemotherapy.

Genomic methods are based on the identification of leukemia-specific genetic aberrations such as chromosomal translocations, point mutations, and gene expression profiles. They help to detect and quantitatively evaluate minimal residual disease.

Nowadays most genomic methods for MRD detection are based on qPCR [35], which achieves an overall sensitivity of 0.001% [18]. PCR amplification of gene rearrangements for MRD detection in ALL has confirmed its significance and has been standardized. However, qPCR use in ALL is limited due to the fact that not all patients have the molecular targets [37]. Some studies demonstrated that consistently negative MRD results received by various methods were associated with very favorable outcomes, consistently positive MRD results were associated with reduced survival, and discrepancies in positive MRD results between methods led to intermediate prognoses. Monitoring MRD with qPCR for detection of gene rearrangements is a sensitive method for predicting outcomes and making treatment decisions after transplantation in patients with ALL. Despite the wide clinical utility and standardization of this method, there have been reports about potential false-positive results caused by massive B-lymphocyte regeneration after HSCT [45].

Currently, MFC and RT-qPCR are the two leading methods for MRD detection. At the same time, MFC with eight–ten color immunostaining is comparable with qPCR in specificity and sensitivity [46]. The assessment with qPCR-MRD is a highly sensitive and standardized method; however, its limitations can provide false-positive results and lead to excessive treatment during the post-transplant period [47].

NGS allows the precise and sensitive detection of multiple antigen receptor rearrangements, thereby providing more specific therapeutic options compared to that of qPCR. The high sensitivity of NGS ensures detection of blast cells not only in the bone marrow but also in the peripheral blood samples [48,49]. Quantitative assessment of MRD with NGS can detect MRD cells at levels ≤0.001%. MRD levels below 0.01% were measured by both qPCR and NGS in a clinical study involving children with ALL who underwent hematopoietic stem cell transplantation [49]. Only one of twenty-seven positive results estimated by qPCR was confirmed as positive by NGS. Interestingly, 10 out of 15 samples that showed low MRD by qPCR in patients who subsequently relapsed were confirmed positive by NGS. These data demonstrate that NGS provides better specificity in ALL evaluation after HSCT, suggesting that therapeutic interventions aimed at eliminating possible relapse should not rely solely on qPCR [47].

The EuroClonality consortium reported a standardized NGS method for identifying targets with IgH and TCR genes [50,51]. This method shows great promise as a replacement for the existing multiplex PCR-based analysis of IgH/TCR gene rearrangements [50]. A recent study has shown that NGS is a more sensitive method in the samples erroneously classified as MRD-negative by MFC-MRD, though, generally, a high correlation is seen between the results received with NGS and MFC or qPCR. The differences are registered in the results of the “grey zone” of low positive values <10^−4^ [52]. Thus, NGS has the potential to be more sensitive than existing MRD detection methods. Theoretically, NGS-based approaches should detect MRD at levels below 10^−5^, with some studies reporting sensitivity down to 10^−7^. It is significantly lower than the achievable level with qPCR-MRD or MFC-MRD. However, this sensitivity requires a large amount of DNA input (i.e., large cell numbers). MRD assessment becomes a challenge in hypocellular samples during therapy cycles [53]. In addition, NGS can be used for simultaneous monitoring of leukemic subclones (intraleukemic heterogeneity), providing alternative data for MRD evaluation in patients with leukemia modulated by immunophenotypic markers undetectable by conventional methods. However, NGS for MRD detection still requires in-depth investigation in larger patient cohorts and development of a standardized approach. Genomic methods have a limitation in terms of the necessity for prior knowledge of the aberrations in the positive sample. Notably, Pulsipher et al. [54] performed a study of NGS-MRD monitoring before and after HSCT, which showed that five out of thirty-eight patients with consistently negative NGS-MRD developed relapses. That may be associated with incomplete sampling of hypoplastic bone marrow of the post-transplantation period.

Thus, NGS-MRD, despite its high sensitivity, has drawbacks as well and can present false-positive and even false-negative results.

Combining methods of MRD detection may not always improve the accuracy of the assessment due to the inconsistency of the results and thus hamper correct interpretation of the received data.

### 3.3. MRD Detection and Clinical Effectiveness

In the late 1990s, the International Berlin–Frankfurt–Münster Study Group (I-BFM-SG) showed that MRD detection on days 33 and 78 of the therapy could distinguish patients with good prognosis (standard risk, MRD-SR) and intermediate prognosis (intermediate risk, MRD-IR) from patients with poor prognosis (high risk, MRD-HR) [55]. The ALL-IC BFM 2002 protocol was the first multicenter randomized clinical trial recommended by I-BFM-SG for MRD assessment by PCR in resource-limited countries. All patients could be classified into risk groups on the basis of the I-BFM-SG results of early peripheral blood response to prednisolone on day 8 and bone marrow blasts assessment on day 15. The next ALL-IC BFM 2009 protocol used only MFC for MRD assessment to introduce a new stratification method.

A Polish retrospective comparative study of the effectiveness of ALL IC-BFM 2002 and ALL IC-BFM 2009 protocols included 3248 patients aged 1 to 18 with newly diagnosed acute lymphoblastic leukemia. The MRD assessment was based on MFC on day 15 of the treatment. The three-year OS for 1872 children treated by the ALL IC-BFM 2002 protocol was 84%, while the OS for 1376 children with ALL treated by the ALL IC-BFM 2009 protocol reached 87%. The corresponding event-free survival (EFS) rates were 82% and 84%, respectively. Although the differences between the effectiveness of the two protocols were not significant, they were statistically valid, and therefore the authors made a conclusion that MRD estimated by MFC on day 15 was an important prognostic factor and that the ALL IC-BFM 2009 protocol improved treatment outcomes for children with ALL compared to the ALL IC-BFM 2002 protocol [13]. On the other hand, a clinical trial including 379 children with ALL and MRD ≥ 10% showed no differences between the effectiveness of the protocols ALL IC-BFM 2002 and ALL IC-BFM 2009 regarding 5-year OS and EFS [56].

The Research Institute of Pediatric Oncology and Hematology, FSBI “N.N. Blokhin National Medical Research Center of Oncology” of the Ministry of Health of Russia, performed a study of the effectiveness of the ALL-IC BFM 2002 protocol. The results showed that complete clinical hematological remission reached 97.9% (*n* = 424 patients) by day 33. The 10-year OS was 91.8 ± 1.5%, and EFS was 84.1 ± 1.9%. The 10-year OS in the groups of standard- and intermediate-risk patients was 92.8 ± 1.7% and 94.6 ± 2.6%, respectively, whereas in high-risk ALL relapse patients, it was 71.1 ± 11.1 [57].

The ALL IC-BFM 2009 protocol was used to treat children with newly diagnosed ALL. The main difference from the previous version, ALL IC-BFM 2002, was MRD detection on day 15 of the therapy as a key criterion for patient stratification into risk groups. In addition, a new stratifying genetic abnormality—hypodiploidy—was included in the protocol. To reduce treatment toxicity, the stage of radiation therapy was abrogated for patients with B-ALL of the HR group stratified into that group only because of poor response on day 8 and patients with T-ALL with initial leukocytosis less than 100 × 10^9^/L without primary CNS lesions. A total of 136 patients were enrolled in the ALL IC-BFM 2009 study from 26 January 2010 to 6 November 2022. Leukemia blast cell tests were performed in all 136 (100%) patients. The cytochemical conclusion for blast cells was based on the negative results of cell staining for myeloperoxidase, nonspecific esterase, chloroacetate esterase, lipids, and PAS-positive reaction. To determine the lineage of ALL, the following markers were used: CD34, TdT, CD19, CD20, CD22, CD79a, cyIgM, CD7, CD5, CD3, CD2, CD4, CD8, CD1a, CD99, CD13, CD14, CD33, CD117, MPO, CD15, CD66c, CD64, CD56, CD61, Glycophorin A, CD38, CD10, HLA-DR, CD123, CD9, NG2, and CD71. The CD34 test was considered positive if more than 10% of malignant blast cells expressed CD34. Other markers were considered positive if they were expressed in 20% of blast cells. Cytogenetic studies of bone marrow samples were performed in 58 (42.6%) patients by karyotyping and fluorescence in situ hybridization (FISH).

MRD levels were determined by flow cytometry on days 15 and 33 of the therapy to monitor the achievement of immunologic remission. The MFC analysis included the following fluorochromes: FITC, PE, PE-Cy5.5, Per-CP, APC, APC-H7, V450, and V500 (Coulter and Sorbent Ltd., Moscow, Russia). Statistical analysis was performed with the program FCS Express v.3. MRD status was defined as “positive” with an outcome of over 0.01% and “negative”—if the outcome was less than 0.01%. Additionally, to monitor molecular remission, MRD levels in three patients were determined by PCR.

In this study, 118 (86.8%) children were diagnosed with B-ALL and 18 patients (13.2%) with T-ALL. Hyperdiploidy and t(12;21)(p13;q22) were the most frequent aberrations revealed in 15/58 (25.9%) and 11/58 (19.0%) patients, respectively. According to the stratification protocol, all children in this study were stratified into three groups by day 33 as follows: 29 (21.3%) patients into the SR group, 79 (58.1%) into the IR group, and 26 (19.1%) into the HR group. Two (1.5%) children were not stratified because of death associated with pancreatic necrosis prior to day 33 and refractory ALL.

Comparison of survival rates in different risk groups showed the highest results in the patients of the SR group; the lowest survival rates were noted in the HR group.

These data demonstrated that eight-color MFC allows a sufficiently accurate identification of MRD. Although FISH tests and karyotyping are commonly used to detect genetic aberrations for the diagnosis and classification of new acute leukemias, these methods are not sensitive enough for MRD monitoring [58]. In general, clinical data show a similar effectiveness of the ALL IC-BFM 2002 and ALL IC-BFM 2009 protocols. Therefore, MFC-MRD and qPCR-MRD have similar prognostic value, although each of them has its drawbacks and can provide false-positive or false-negative results. Ștefan AI et al. [58] performed a meta-analysis of 13 clinical trials to compare the prognostic and predictive effectiveness of MFC or qRT-PCR and NGS. The results showed a high concordance between positive MFC-MRD and NGS-MRD tests (79.9–97%). However, NGS revealed 18–30% of MRD-positive cases that were not detected with MFC. Patients with negative MRD-MFC but positive MRD-NGS had poorer prognoses than those with negative MRD-MFC and MRD-NGS tests [59]. These data demonstrate that despite the high sensitivity of MRD-MFC and MRD-PCR, which allows patient risk stratification and beneficial clinical outcomes with protocols ALL IC-BFM 2002 or ALL IC-BFM 2009, these tests may present false-negative results and affect the choice of treatment strategy. The NGS test, which is more sensitive, may detect MRD in cases with negative MFC and qRT-PCR results. However, highly sensitive NGS tests can lead to false-positive results since not all MRD-NGS-positive or MRD-MFC-negative patients developed a relapse.

## 4. Discussion

MRD is an integral part of the treatment of patients with acute leukemia and other hematological malignancies. In addition to risk stratification for cancer relapse, MRD is used for making decisions about hematopoietic cell transplantation, pre-transplant conditioning, and/or duration of post-transplant interventions such as immunosuppression and/or preventive post-transplant anti-leukemic therapy [60,61,62,63,64]. Despite numerous studies and recommendations, MRD is still an unresolved issue. Nowadays the most common diagnostic method is MFC-MRD, providing the necessary threshold sensitivity of 10^−4^ with standard four–six colors. The EuroFlow consortium protocol recommends eight-color MFC-MRD that can reach sensitivity up to 10^−5^. Our clinical research, conducted according to the ALL-IC BFM 2009 protocol, demonstrated high clinical efficacy, confirming its prognostic significance. However, some leukemic clones may alter their antigen profile during chemotherapy. The heterogeneity of transformed cells can lead to false-negative results, thereby limiting the MFC effectiveness. Furthermore, MFC-MRD can show false-negative results after HSCT during the period of hematopoietic recovery while the immunophenotype of leukemic blasts significantly overlaps with that of hematogones or normal B-cells. An important factor for successful flow cytometry is the number of target cells: at least 4 million, which is difficult to achieve due to suppression of lymphopoiesis during chemotherapy. A significant drawback of classical MFC-MRD is the lack of standardization of immunophenotyping protocols and antibody panels.

To identify recurrent fusion gene transcripts (e.g., BCR::ABL1 and E2A::PBX1) in B-ALL, PCR is used to achieve sensitivity higher than 10^−5^ [47]. Both the implementation of up-to-date sequencing methods and the discovery of new genetic alterations (e.g., IKZF1 and PAX5) significantly expanded the MRD detection on the basis of genetic changes. Discrepant results of MFC and PCR analyses were noted in 5 to 20% of samples [48]. Although negative MRD results obtained by MFC and PCR correlate with favorable outcomes, these patients can develop relapses in 25–40% of cases [9,65,66].

Regarding diagnosis and treatment of hematological malignancies, NGS has a higher sensitivity and accuracy for MRD monitoring than MFC or qPCR [67]. Monitoring MRD by sensitive and reliable NGS improves the detection of relapse risk after HSCT or CAR-T-cell therapy. Negative NGS-MRD can identify a group of patients with B-ALL at low risk of relapse. This is a more advanced method of prediction than MFC-MRD. The sensitivity of NGS-MRD is ≥10^−6^, and the results correlate with relapse and poor survival. This method revealed a false-positive MRD received with PCR [47]. However, the interpretation of NGS-MRD results has limitations that should be considered while making treatment decisions for children with ALL. Nevertheless, this highly sensitive method of MRD detection may provide false-negative results since some patients lack identifiable clones at the time of diagnosis [67,68,69,70,71]. Thus, NGS-MRD may be unfeasible, and the results of IgH clonality may not be available for targeted patients. In this case, MFC-MRD seems more reliable [14].

Any method of quantitative MRD assessment can yield conflicting results, making their interpretation a real challenge. Particularly, MRD detection by MFC and qPCR in the same sample can provide different outcomes [64]. That means that different methods for MRD detection can lead to false-positive and false-negative results [24]. On the one hand, false-negative results impede effective therapeutic strategies. On the other hand, false-positive MRD may lead to excessive treatment and increasing severity of toxicity. Therefore, it is important to avoid both false-positive and false-negative MRD results [58]. An essential issue is defining the clinically significant MRD level and the choice of an appropriate method. So far, the reports have presented controversial data when discussing whether sensitivity levels over 10^−4^ provide advantages in MRD assessment and if single transformed cells detected with highly sensitive methods of 10^−5^ to 10^−7^ can develop a relapse [72].

One of the ways to increase the accuracy of MRD detection at the required levels may be additional control using cell sorters for positive target cells and standard cytomorphological staining.

## 5. Conclusions

At present, the most commonly used method for MRD detection provides the necessary sensitivity of 10^−4^ and ensures reliable prediction of ALL dynamics and effective therapeutic strategies. However, even multicolor flow cytometry cannot avoid cases of false-positive and false-negative results. Therefore, application of highly sensitive and highly productive genomic methods in addition to MFC may enhance the accuracy of MRD assessment. On the other hand, overwhelming efforts to reach the highest sensitivity of the detection methods may lead to the detection of clinically insignificant manifestations of minimal residual disease and, subsequently, to unjustified escalation of antitumor therapy. Thus, there is a challenge to find the necessary ground for an adequate sensitivity of the MRD detection methods that could ensure the fine line between false-positive and false-negative MRD results in patients with childhood ALL to develop an appropriate therapeutic strategy.

## Figures and Tables

**Table 1:** Major characteristics of methods for MRD detection.

Parameter	Flow Cytometry	Polymerase Chain Reaction	Next-Generation Sequencing
Object of the study	Blasts with the aberrant immunophenotype	Tumor-specific transcript and molecular rearrangements	Multiple antigen receptor rearrangements
Sensitivity	10^−3^–10^−5^	10^−4^–10^−6^	10^–5^–10^−7^
Advantages	(1) The possibility of using in most cases (2) Fast execution (3) The possibility of obtaining additional data on a malignant or non-malignant cell population	(1) High sensitivity (2) DNA stability (3) A high level of standardization (4) Availability of the accumulated data to rely on when choosing a treatment strategy	(1) More sensitive than PCR (2) Better specificity after HSCT (3) Tracking multiple sequences with unprecedented sensitivity (4) Possibility for simultaneous monitoring of multiple leukemic subclones in the same patient
Disadvantages	(1) The result can be affected by increasing the pool of cell precursors during regeneration, low cell content of the samples, or altered immunophenotype during/after induction therapy (2) Limited sensitivity	(1) Long-term processing (2) High cost (3) The result can be affected by clonal evolution	(1) Few studies of the NGS feasibility in terms of MRD detection (2) Excessive DNA input is necessary (3) The requirement to know the initial aberrations in the positive sample

HSCT—hematopoietic stem cell transplantation, PCR—polymerase chain reaction, NGS—next-generation sequencing, MRD—minimal residual disease.

## Abbreviations

The following abbreviations are used in this manuscript:

ALL	Acute lymphoblastic leukemia
AML	Acute myeloid leukemia
B-ALL	B-lineage acute lymphoblastic leukemia
CAR-T	Chimeric antigen receptors (chimeric T-cell receptors)
CNS	Central nervous system
DfN	Difference from normal
dPCR	Digital PCR
EFS	Event-free survival
FC	Flow cytometry
FISH	Fluorescence in situ hybridization
HSCT	Hematopoietic stem cell transplantation
I-BFM-SG	International Berlin–Frankfurt–Münster Study Group
IgH	Immunoglobulin H
LAIPs	Leukemia-associated immunophenotypes
MFC	Multicolor flow cytometry
MRD	Minimal residual disease
MRD-HR	Minimal residual disease—high risk
MRD-IR	Minimal residual disease—intermediate risk
MRD-SR	Minimal residual disease—standard risk
NGS	Next-generation sequencing
OS	Overall survival
PAS	Periodic acid Schiff
PCR	Polymerase chain reaction
qPCR	Quantitative PCR
RFS	Relapse-free survival
T-ALL	T-lineage acute lymphoblastic leukemia
TCR	T-cell receptor
